# Pulmonary Embolism Secondary to Testosterone-Enhancing Herbal Supplement Use

**DOI:** 10.7759/cureus.1545

**Published:** 2017-08-06

**Authors:** Steven M Nguyen, Nway Ko Ko, Asad S Sattar, Esra Gucuk Ipek, Sayed Ali

**Affiliations:** 1 Department of Civil Engineering, Carnegie Mellon University; 2 Internal Medicine, University of Central Florida College of Medicine; 3 Medicine, Orlando VAMC

**Keywords:** pulmonary embolism, testosterone, herbal medicine

## Abstract

Decreased testosterone levels in men are often a normal sign of aging. Testosterone replacement therapy (TRT) is a well-established option for those with symptomatic hypogonadism related to low testosterone levels. Conversely, designer herbal supplements in the context of testosterone supplementation are poorly studied, yet remain popular among aging men who seek the well-known, often enhancing, effects of testosterone that involve muscle mass and sexual function/drive. In 2014, the Food and Drug Administration (FDA) issued a warning about the significant risk of venous clots secondary to testosterone product use. Testosterone-induced polycythemia is one of the proposed mechanisms for this increased clotting propensity. Increased thromboxane A2 receptor density on platelets and increased platelet aggregation have also been linked to testosterone treatment in men. Fenugreek extract is a common active ingredient in commercially available herbal supplements that are often marketed as testosterone enhancers. It is thought that certain fenugreek compounds inhibit aromatase and 5-alpha-reductase activity, leading to diminished testosterone breakdown. However, the efficacy and safety profile of this agent in its use for boosting testosterone levels are unclear. In this case report, we present a patient with new-onset, bilateral pulmonary embolism possibly associated with the daily use of fenugreek-containing testosterone supplements.

## Introduction

The benefits of testosterone replacement therapy (TRT) are well-established for men with an age-related decline in testosterone and evidence of pathological hypogonadism [1]. The known effects of testosterone on sexual libido and function in addition to lean body mass have popularized over-the-counter hormone supplementation in aging men, even in those without a clinical need for TRT. As a result, there is an increasing interest in dietary or herbal supplements that are marketed as testosterone-boosting drugs. Despite their popularity, over-the-counter testosterone-boosting products have been poorly studied and are loosely regulated. One example of such supplementation involves extracts from the fenugreek plant. Fenugreek (*Trigonella foenum-graecum)* is commonly listed as an active ingredient in these designer testosterone enhancers [2]. The mechanism of the action of fenugreek extract on testosterone levels is unclear. It has been proposed to act through aromatase and 5-alpha-reductase inhibition, leading to decreased testosterone breakdown to estrogen and dihydrotestosterone end products [3]. The quality of the available basic science data is low, however.

In contrast to its benefits, the adverse effects of testosterone replacement and enhancement products are not as widely publicized. There is an established association between testosterone therapy and cardiovascular events [4]. The specific mechanisms behind this are still not well-defined; however, several mechanisms have been proposed and studied [4]. Testosterone can affect erythropoietin production, which can ultimately increase the risk of polycythemia through erythropoietic stimulatory effects [5]. Vascular events, such as pulmonary embolism (PE), are potential complications of polycythemia [5]. Testosterone therapy has also been associated with increased thromboxane A2 receptor expression on platelets in addition to increased platelet aggregation, potentially creating a higher risk for thrombus formation [4]. In this report, we present a case of new-onset, bilateral PE secondary to over-the-counter fenugreek-extract-containing testosterone supplements.

## Case presentation

A previously healthy 51-year-old man presented in urgent care with a two-day history of left-sided chest pain radiating to the neck. The pain worsened with inspiration and was not alleviated with over-the-counter ibuprofen. He denied having a fever, nausea, recent infection, cough, hemoptysis, calf pain, or swelling. He did not have any recent infections, recent trauma, prolonged immobilization, or recent travel. He denied any deep vein thrombosis, PE, and hypercoagulable diseases. There was no family history of hypercoagulable disease either. He had never smoked cigarettes or used illicit drugs. He began the daily use of testosterone supplements three months ago in the hope of increasing muscle mass but stopped taking these supplements one week ago. He also reported chronic creatine monohydrate supplementation from commercially available products.

**Figure 1:**
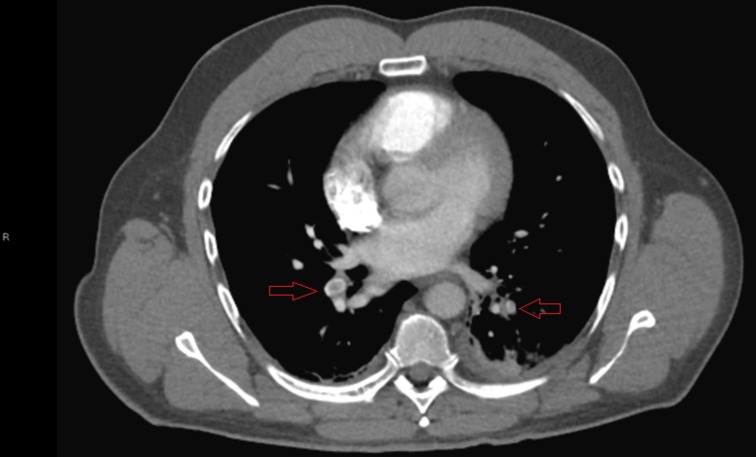
Computed tomography angiography demonstrating thrombi in lower lobe segmental arteries bilaterally

On arrival, he was afebrile, tachycardic, and normotensive, and displayed normal breathing. On auscultation, his lungs were clear bilaterally; the cardiac exam revealed tachycardia but an otherwise regular rhythm. There was no jugular vein distention, organomegaly, murmurs, or lower-extremity edema appreciated. The rest of his physical exam was unremarkable. His laboratory tests revealed elevated D-dimer (1.93 mcg/mL) and leukocytosis (15.5 K/cmm). Troponin levels were within normal limits. A computed tomography (CT) angiography ultimately revealed bilateral lower-segmental PEs, as seen in Figure 1. An electrocardiography, a chest X-ray, and a Doppler ultrasound of the lower extremities were unremarkable. He received an initial dose of enoxaparin and was subsequently started on rivaroxaban. Further laboratory workup showed that his total testosterone level was low. His three-day hospital course was complicated by persistent shortness of breath, left-sided chest pain, and intermittent oxygen desaturation (range 88–92%), all worsened by even minimal exertion (e.g., walking). An echocardiography was performed, and it was unremarkable; there was normal right ventricular function and size. He was finally discharged with anticoagulation therapy and home oxygen supplementation after an unexpectedly prolonged stay.

The etiology of his PEs was ultimately attributed to his recent testosterone supplementation. The patient presented with no other plausible risk factors for abnormal clot formation. We advised the patient to discontinue his testosterone supplement intake as well as to avoid other commercial supplements.

## Discussion

 

In 2014, the Food and Drug Administration (FDA) issued a general warning for the risk of venous blood clots associated with testosterone product use [6]. The FDA warning specifically referenced this risk as a potential consequence of testosterone-induced polycythemia although the exact pathophysiology behind the association between testosterone therapy and vascular events is still unclear. A randomized, placebo-controlled trial by Ajayi et al. demonstrated a significant association between increased platelet thromboxane A2 receptor density and testosterone therapy in otherwise healthy male patients [7]. The molecular mechanism behind this result was not elucidated in the study; however, the authors suggest that testosterone likely regulates the expression of thromboxane A2 receptors on platelets. These investigators also found a significant increase in platelet aggregation in follow-up assays when compared to the study’s placebo arm [7]. A retrospective, population-based case-control study of 928,745 patients showed a significantly increased incidence rate of venous thromboembolism within six months of testosterone therapy use in men without a prior history of venous thromboembolic risk; these patients were prescribed a testosterone therapy regimen of at least a 30-day duration (rate ratio: 1.88, 95% CI 1.02–3.45) [8].

The timing of our patient’s diagnosis with his use of designer testosterone-enhancing supplements was of concern, especially given the lack of any hypercoagulable risk factors. Additionally, he was found to have low serum testosterone levels on admission. The significance of testosterone levels has not been well-established in previous studies assessing the association between testosterone therapy and thrombotic vascular events [4]. Furthermore, testosterone levels in men receiving supplemental treatments have been shown to return to below-baseline pretreatment levels as early as two weeks after the last administration and following a transient increase in testosterone levels [7], which may explain the low levels in our patient. There is also limited evidence involving the mechanism by which our patient’s particular herbal supplement increases testosterone levels. The toxicity profile of the fenugreek-containing product in question is not well-established either.

Similar cases have been documented involving PEs and the use of testosterone supplements. Bui et al. reported on a 65-year-old man who was hospitalized for bilateral PE with confirmed deep vein thrombosis [9]. He had been taking diindolylmethane (DIM) for three months. DIM is another poorly studied commercial plant extract that is thought to act on androgen physiology; however, much like with fenugreek, the exact effects are unclear. Unlike our patient, however, venous thromboembolism risk factors were readily identified: a history of tobacco use, obesity, age, and a suspected prior PE.

Another interesting case series by Glueck et al. described unprovoked significant thrombotic events (PE, amaurosis fugax, and osteonecrosis of the hips) in men with previously undiagnosed thrombophilias receiving clinically prescribed exogenous testosterone [10]. The authors suspected that the recent thrombotic events were multifactorial in etiology but were primarily attributed to the risk for clotting propensity from TRT superimposed on the newly diagnosed underlying hypercoagulable profile. Ultimately, the true etiology behind our patient’s PE was not definitively elucidated from the workup during his hospital stay; however, suspicion is high for this understudied herbal testosterone supplement, especially given the historical evidence of the association between recent testosterone therapy and venous thromboembolic events.

## Conclusions

There is an inherent risk for vascular events, such as pulmonary embolus, in testosterone supplement use. The mechanism behind this risk is unclear; however, there is evidence of an association between testosterone therapy and increased platelet aggregation, increased thrombus-promoting receptor expression on platelets, and polycythemia. The risk of venous thromboembolic events is even more unclear with non-FDA-approved herbal supplements marketed as testosterone enhancers. Our report demonstrates a case of such potential consequences. Men without pathological hypogonadism who express concern over testosterone levels should be cautioned on the potential adverse effects and risks of self-experimentation with over-the-counter testosterone products.
